# Thermally Rearranged Mixed Matrix Membranes from Copoly(o-hydroxyamide)s and Copoly(o-hydroxyamide-amide)s with a Porous Polymer Network as a Filler—A Comparison of Their Gas Separation Performances

**DOI:** 10.3390/membranes12100998

**Published:** 2022-10-14

**Authors:** Cenit Soto, Bibiana Comesaña-Gandara, Ángel Marcos, Purificación Cuadrado, Laura Palacio, Ángel E. Lozano, Cristina Álvarez, Pedro Prádanos, Antonio Hernandez

**Affiliations:** 1 Surfaces and Porous Materials (SMAP), Associated Research Unit to CSIC, Facultad de Ciencias, University of Valladolid, Paseo Belén 7, 47011 Valladolid, Spain; 2 Institute of Sustainable Processes (ISP), Dr. Mergelina s/n, 47011 Valladolid, Spain; 3 Institute for Polymer Science and Technology (ICTP-CSIC), Juan de la Cierva 3, 28006 Madrid, Spain; 4 Department of Organic Chemistry, School of Sciences, University of Valladolid, Paseo Belén 7, 47011 Valladolid, Spain; 5 IU CINQUIMA, University of Valladolid, Paseo Belén 5, 47011 Valladolid, Spain

**Keywords:** CO_2_ separation, mixed matrix membranes, porous polymer networks, thermal rearrangement

## Abstract

Copoly(o-hydroxyamide)s (HPA) and copoly(o-hydroxyamide-amide)s (PAA) have been synthesized to be used as continuous phases in mixed matrix membranes (MMMs). These polymeric matrices were blended with different loads (15 and 30 wt.%) of a relatively highly microporous porous polymer network (PPN). SEM images of the manufactured MMMs exhibited good compatibility between the two phases for all the membranes studied, and their mechanical properties have been shown to be good enough even after thermal treatment. The WAX results show that the addition of PPN as a filler up to 30% does not substantially change the intersegmental distance and the polymer packing. It seems that, for all the membranes studied, the free volume that determines gas transport is in the high end of the possible range. This means that gas flow occurs mainly between the microvoids in the polymer matrix around the filler. In general, both HPA- and PAA-based MMMs exhibited a notable improvement in gas permeability, due to the presence of PPN, for all gases tested, with an almost constant selectivity. In summary, although the thermal stability of the PAA is limited by the thermal stability of the polyamide side chain, their mechanical properties were better. The permeability was higher for the PAA membranes before their thermal rearrangement, and these values increased after the addition of moderate amounts of PPN.

## 1. Introduction

In recent decades, membrane science and technology has attracted great interest at both academic and industrial levels as the cornerstone of sustainable processes with lower environmental impacts and higher efficiencies. The separation, concentration and purification of chemical species present in gas and liquid mixtures constitute a technological challenge in the chemical, petrochemical, biological, pharmaceutical, food and beverage and environmental sectors [1], among others. In this context, conventional techniques such as cryogenic distillation and absorption, which involve a phase change with subsequent energy consumption, have been displaced by membrane-based separation processes, which are much faster, efficient and economical [2,3,4]. In the particular case of gas separation and purification applications, membranes represent an energy-efficient alternative with a small carbon footprint, an easy scale-up and environmental friendliness [4,5,6]. The selection of membrane materials in gas separation applications is based on the chemical and physical properties of the target gases and membrane material. Indeed, gas separation depends on the interaction between the gas molecules and the material, structure, thickness and configuration of the membrane, as well as the modulus and system design [5]. In this regard, commercial membranes must exhibit easy processing, high flexibility, low cost and mechanical strength [2].

The development of new materials for gas separation aims to increase the permeability without compromising the selectivity, or vice versa. The existence of an inverse relationship between these two variables (permeability and selectivity) is one of the main disadvantages of polymeric membranes. To overcome this trade-off relationship between permeability and selectivity, multiple investigations have been carried out to develop new materials to separate different gas mixtures such as O_2_/N_2_ from air, CO_2_/CH_4_ from landfills, biogas or natural gas, CO_2_/N_2_ in power plants, etc. [7]. In this context, Mixed Matrix Membranes (MMMs) have emerged as a new means to increase gas separation efficiency. These MMMs are the result of the addition of organic and inorganic materials (disperse phase) to the polymers (continuous phase) in order to form a hybrid material [8]. In recent years, a wide variety of fillers and polymers have been investigated to manufacture MMMs that are able to increase permeability while maintaining selectivity. For instance, the combination of the 6FDA-DAM polymer and the ZIF-94 filler results in an increase in CO_2_ permeability while maintaining a constant selectivity for the CO_2_/N_2_ gas pair [9]. Likewise, PIM-1 as a polymeric matrix loaded with the metal-organic frameworks UiO-66, UiO-66-NH_2_ and UiO-66-(COOH)_2_ showed a good performance in removing CO_2_ from biogas and flue gas [10] due to their high permeability for the single gases CH_4_ and CO_2_ and good selectivity in the binary mixture CO_2_/CH_4_. Polyimides such as 6FDA−6FpDA, 6FDA−TMPD and Matrimid^®^, combined with Porous Polymer Networks (PPNs) fillers, based on triptycene-isatin and 1,3,5-triphenylbenzene-4,5-diazaflouoren-9-one, have produced MMMs showing a high gas permeability together with a selectivity for CO_2_/N_2_ and CO_2_/CH_4_ gas pairs, similar to the matrix membranes [11,12,13]. On the other hand, thermal rearrangement seems to be a good additional improvement, leading to increasing permeability, but also because plasticization is significantly reduced [14].

PPNs are quite interesting for gas separation due to their high thermal and chemical stability, easy processing and low cost [15]. PPNs are synthesized by the homocoupling of tetrahedral monomers via the oxidative Eglinton coupling or Yamamoto-type Ullmann coupling reaction, exhibit high thermal and chemical stability and are insoluble in conventional solvents. PPNs possess very high BET areas. Recently, polymers and copolymers capable of producing poly-1,3-benzoxazoles when subjected to a thermal rearrangement, such as ortho-hydroxy polyimides and polyamides, have been used as a polymeric matrix to manufacture Thermally Rearranged MMMs (TR-MMMs). For example, TR-MMMs manufactured by the incorporation of a porous polymer network, PPN, based on triptycene and trifluoroacetophenone into a polyamide, 6FCl-APAF, showed significant improvements in permeability values and a slight decrease in the selectivity relative to the neat polyamide [16] such that they surpassed the 1991 Robeson limit for the CO_2_/CH_4_ gas pair. Similarly, aromatic ortho-hydroxypolyimide (PIOH) and ortho-acetylpolyimide (PIOAc) loaded with PPNs based on triptycene-isatin as a filler were evaluated as TR-MMMs and supported an increase in CO_2_ gas permeability [17] that even surpassed the 2008 Robeson limit. Finally, PAF-1 nanoparticles were added into the TR-able 6FDA-HAB5DAM5 (DAM) copolymer, with subsequent improvements in H_2_ and CO_2_ transport and selectivity enhancement for H_2_/N_2_, H_2_/CH_4_ and CO_2_/CH_4_ separations [18].

In this work, the use of triptycene-isatin PPNs as a filler and copolyamides as polymeric matrixes capable of producing thermally rearranged polybenzoxazoles (TR-PBOs) to obtain TR-MMMs has been tested. The copolyamides are derived from the combination of TR-able diamines (4’4-propane-2,2-diylbis(2-aminophenol), APA, 2,2-bis(3-amino-4-hydroxyphenyl)hexafluoroisopropylidene, APAF) and non-TR-able diamines (4,4’-(hexafluoroisopropylidene)dianiline, 6FpDA) with 2,2-bis(4-chlorocarbonylphenyl)hexafluoropropane (6FCl). These copolyamides—copoly-o-hydroxyamides (APAF-APA-6FCl) and copoly-o-hydroxyamide-amides (APAF-6FpDA-6FCl)—will be referred to as HPA and PAA, respectively. The benefit of the combination of these two materials was herein assessed by determining the transport properties of the formed mixed matrix membranes before (MMMs) and after thermal rearrangement (TR-MMMs). Therefore, this work aimed to demonstrate the enhanced performance of the filler over TR-able copolymers as a strategy to improve the membrane performance for gas separation.

## 2. Experimental

### 2.1. Materials

Anhydrous N,N-Dimethylacetamide (DMAc, purity 99.8%), N,N-dimethylaminopyridine (DMAp, purity ≥ 99.0%), Chlorotrimethylsilane (CTMS, purity 98.0%), Tetrahydrofuran (THF, purity 99.9%) and pyridine (Py, purity 99.8%), were purchased from Sigma-Aldrich (Saint Louis, MO, USA) and were used without additional purification in order to prepare the target aromatic polyamides. Thionyl chloride (SOCl_2_), distilled under reduced pressure before use, N,N-Dimethylformamide (DMF, purity 99.8%), N- methyl-2-pyrrolidinone (NMP, purity 99.5%), 4,4′-(hexafluoroisopropylidene)bis(benzoicacid) (6F, purity 98%) and trifluoromethanesulfonic acid (TFSA, purity 98%) were bought from Sigma-Aldrich. Methanol (MeOH) reagent-grade quality, used without purification, was purchased from Scharlab (Barcelona, Spain). Isatin (1H-indole-2,3-dione; Aldrich, 99%) and trypticene (ABCR, purity 99%) were employed as received. The diamines 2,2-bis(3-amino-4-hidroxyphenyl)hexafluoroisopropylidene (APAF, purity ≥ 99.0%) and 4,4’-(hexafluoroisopropilidene)dianilina (6FpDA, purity ≥99.0%) were purchased from Apollo Scientific (Manchester, UK) and purified by sublimation under reduced pressure at 220 °C and 180 °C, respectively, just before use. 4’4-Propane-2,2-diylbis(2-aminophenol) (APA, purity ≥ 97.0%) was also purchased from Apollo Scientific and purified by recrystallization from hot methanol. 

### 2.2. Synthesis of 6FCl Acid Dichloride

The diacid chloride 6FCl was synthesized using the reaction of its corresponding diacid, 6F, following the detailed procedure from Soto et al. [16]. The diacid was mixed with SOCl_2_ in a reflux system under constant agitation in the presence of some drops of DMF (as a catalyst) at 50 °C for 4 h followed by 80 °C for 2 h to complete the reaction. The reaction vessel was allowed to cool down before distilling the SOCl_2_ and DMF in a distillation column. Toluene was added in order to eliminate the residual thionyl chloride and to avoid the drying of the distillation column [19]. The obtained diacid chloride, 2,2-bis(4-chlorocarbonylphenyl)hexafluoropropane (6FCl), was crystallized from cool hexane while keeping the solution at 0 °C overnight. Finally, 6FCl was purified by sublimation at 120 °C.

### 2.3. Synthesis of Polyamides

**Figure 1:**
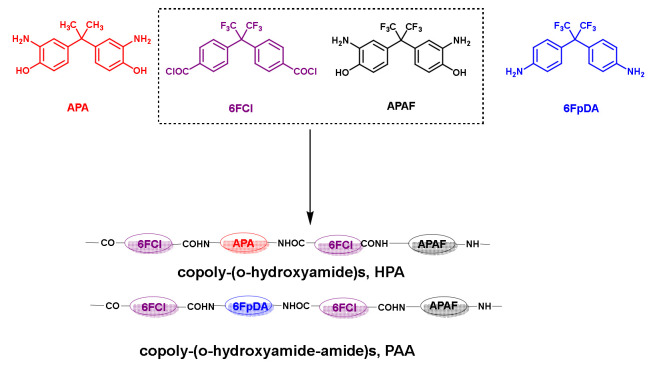
General polymerization reaction for copoly-*o*-hydroxyamides and copoly-*o*-hydroxyamide-amide.

The synthesis of copoly-o-hydroxyamide-amide (PAA) and copoly-o-hydroxyamide (HPA) was carried out by polycondensation at a low temperature, employing the in situ diamines silylation method following the procedure described by Soto et al. [16], with a co-base (DMAP) as an activation agent. The diamines used were APA, APAF and 6FpDA in combination with 6FCl. The combination of 6FCl and APAF with 6FpDA or APA resulted in the formation of two new aromatic copolyamides, obtained following the general procedure described below (Figure 1). Briefly, the x and y mmoles (x + y = 10 mmoles) of the two corresponding diamines were dissolved in 5 mL of DMAc at room temperature in a three-necked flask under an inert atmosphere equipped with a mechanical stirrer. The reaction was cooled down in an ice bath to 0 °C before the slow addition of 22 mmoles of CTMS followed by 20 mmoles of Py. The ice bath was removed, and the reaction mixture was stirred at room temperature for 15 min to ensure the silylation of the diamines. The reaction was again cooled to 0 °C, and 10 mmol of diacid chloride and 5 mL of DMAc were then added. Finally, 2 mmol (10% mol/mol pyridine) of DMAP was added. The reaction mixture was kept under agitation at room temperature for 24 h. The viscous polymer solution was precipitated in water and then rinsed in an ethanol–water mixture. The polymer fibers were dried at 80 °C overnight without vacuum and then at 100 °C under vacuum for 24 h. The resulting co-polymers, named APAF-6FpDA-6FCl (PAA) and APAF-APA-6FCl (HPA), were used as the polymeric matrix for the manufacture of MMMs and their corresponding TR-MMMs.

The polymers were characterized by nuclear magnetic resonance spectroscopy (NMR, ^1^H) using Varian AV Agilent (Agilent Tech., Santa Clara, CA, USA) equipment operated at 400 MHz for 1H and 75 MHz for ^13^C. The assignation of the structures of the polymers synthesized was reported in a previous work [20].

The estimation of the molecular weight of the two prepared copolyamides was carried out by determining the inherent viscosities in an automatic Ubbelohde viscometer at 25 °C using 0.5% (0.5 g/dL) polymer concentrations in NMP. Each polymer solution was measured six consecutive times, and an average value of the viscosity was calculated. It is known that the in situ silylation of the aromatic gives high-molecular-weight polyamides [20,21]. In this context, the inherent viscosity (ƞ_inh_) of our polymers was 1.65 (dL/g) for APAF-6FpDA-6FCl and 1.00 (dL/g) for APAF-APA-6FCl, which indicates the high molecular weight of the polymers synthesized. These polyamides were soluble at room temperature in polar aprotic solvents such as dimethyl sulfoxide (DMSO), NMP, DMAc and THF. According to a previous work [22], THF was selected as a solvent to prepare the MMMs because it does not generate particle agglomeration of the filler during membrane manufacture.

### 2.4. Synthesis of the Porous Polymer Network as a Disperse Phase

**Figure 2:**
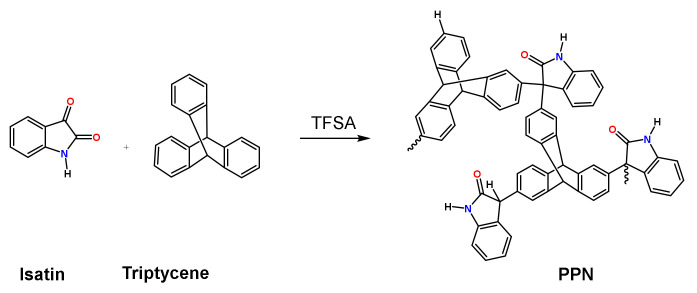
PPN derived from triptycene and isatin.

The PPN was synthesized by combining isatin and trypticene following the synthesis method described by López-Iglesias et al. [13]. Thus, trypticene, isatin and chloroform were added in a three-necked Schlenk flask equipped with a mechanical stirrer at room temperature and under an N_2_ blanket. The mixture was cooled to 0 °C before slowly adding TFSA. Then, the reaction was maintained under mechanical stirring for 5 days at room temperature. The product obtained was poured into a water–ethanol mixture (3/1) and filtered. Afterwards, it was washed with water, acetone and chloroform and acetone and water. Finally, it was dried at 150 °C for 12 h under vacuum. A brown powder material with a 98% yield was obtained (Figure 2).

The characterization of PPNs in terms of chemical structure (by FTIR), porosity (by BET, surface) and thermal stability (by TGA) was carried out as reported elsewhere [13]. The absorption bands at 1708, 1469 and 1320 cm^−1^ in the FTIR spectra were attributed to the five lactam rings (see Figure 2). The low-pressure N_2_ adsorption isotherm at −196 °C (77K) showed that the PPN was a highly microporous material with a specific surface area (SBET) of 790 m^2^ g^−1^ and a total pore volume of 0.44 cm^3^ g^−1^, while the CO_2_ adsorption isotherms at 0 °C revealed a high volume of pores < 0.7 nm (0.36 cm^3^ g^−1^). Moreover, the PPN showed CO_2_ uptakes of 207 mg g^−1^/4.70 mmol g^−1^. The wide-angle powder X-ray diffraction analysis confirmed the amorphous nature of the PPN, which also presented a high thermal resistance in N_2_ with an onset of the degradation temperatures above 450 °C.

### 2.5. Membrane Manufature

The HPA-PPN and PAA-PPN MMMs were manufactured by the casting of polyamide (HPA or PAA) solutions containing different loadings of PPN, followed by slow solvent evaporation, according to the procedure reported elsewhere [16]. The polymer solution (continuous phase) was prepared by dissolving 1 g of the dried polymer in 10 mL of the THF as a solvent. Separately, the required amount of PPN (disperse phase) was calculated according to Equation (1). Thus, 15 and 30 wt.% of the filler to the polymer matrix were dispersed in THF under constant stirring for 24 h. To ensure an effective dispersion of the particles, the suspension was subjected to sonication using a Vibra Cell 75,186 (Sonics, Newtown, CT, USA) at 30% of amplitude for 20 min (40 cycles of 20 s ultrasonic exposures and 10 s cool-downs). Additionally, the priming strategy was employed to reduce stress at the polymer–particle interface, decrease particle agglomeration [23,24] and improve the good compatibility between the filler and the polymeric matrix. Approx. 10% of the polymer solution was added to the PPN particles suspension that was sonicated again for 10 min prior to adding the rest of the polymer. After stirring for 2 h, the homogeneous suspension was casted on a leveled glass plate and covered with a watch glass to allow for a slow evaporation of the solvent at room temperature overnight, followed by 60 °C (12 h), 80 °C (2 h) and, finally, vacuum drying at 100 °C (2 h), 120 °C (2 h), 150 °C (1 h) and 180 °C (12 h). Neat membranes, which will be used as reference materials, were casted following the same procedure. The thicknesses of the resulting films ranged between 40 and 50 μm. The load is evaluated according to Equation (1).

(1) $$PPN(\%)=\;\frac{filler\;weight}{filler\;weight+\;polymer\;weight}\;\times \;100$$

### 2.6. Thermal Rearrangement

**Figure 3:**
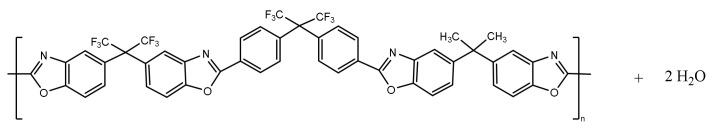
Result of the thermal rearrangement of HPA to HPA-TR plus water. See Figure 1 for the structure of HPA.

HPA and PAA copolyamides were converted to polybenzoxazoles or poly(amide-benzoxazoles) by subjecting them to the thermal treatment described below. The reaction scheme from HPA to TR-HPA is shown as an example in Figure 3.

The MMMs and neat membranes were sandwiched between two quartz plates to avoid film rolling at high temperatures before being placed in a Carbolite CTF 12/65/700 (Carbolite-Gero, Hope, Derbyshire, UK) under an N_2_ atmosphere (0.3 L min^−1^) pyrolizer furnace. The samples were subjected to the following thermal protocol: heating up to 150 °C at a rate of 10 °C/ min (15 min of dwell time), heating to 250 °C at a rate of 5 °C/min (30 min of dwell time) and, finally, heating to 375 °C at a rate of 5 °C/min (15 min of dwell time). Finally, the furnace was cooled to room temperature at 10 °C/min. The temperatures and heating rates were previously optimized by means of thermogravimetric studies. The acronyms of the manufactured membranes are shown in Table 1. Note that we only included up to 30% PPN content, because previous experience revealed that PPN content over 30% strongly decreases the selectivity and worsens the mechanical properties [25].

To quantify the conversion percentage from HPA and PAA to TR-PBO, Equation (2) was used.

(2) $$\%conversion\;=\;\frac{Actual\;mass\;loss}{Theoretical\;mass\;loss}\;\times 100$$

where the ‘‘actual mass loss’’ is the mass loss observed via thermo-gravimetric analysis (TGA), and the ‘‘theoretical mass loss’’ is the mass loss expected if the reaction shown in 3 was to proceed to completion [26].

### 2.7. Characterization of the Membranes

To identify the main functional groups of components of the MMMs and the resulting TR- MMMs, Attenuated Total Reflectance-Fourier Transform Infrared Spectroscopy (ATR-FTIR) was carried out using a PerkinElmer Spectrum One FT-IR (PerkinElmer, Waltham, MA, USA) equipped with a Universal ATR diamond-tipped sampling accessory module.

The thermal behavior of the films was assessed by thermogravimetric analysis (TGA) using a TA-Q-500 thermogravimetric analyzer (TA Instruments-Water Corp. Milford, MA, USA). All TGA experiments were carried out under nitrogen atmosphere (60 mL min^−1^), with 3–6 mg samples subjected to a final temperature of 800 °C. Isothermal TGA was conducted using the following thermal protocol in order to elucidate the conversion percentage of the TR-PBO-MMMs: the sample was heated at 250 °C at 5 °C min^−1^, held at this temperature for 30 min and then heated up to the rearrangement temperature (375 °C) at 5 °C min^−1^ and held for 2 h.

Differential Scanning Calorimetry (DSC) was carried out using a TA Instruments DSC Q-20 Analyzer to monitor the glass transition temperatures (Tg) (TA Instruments-Water Corp., Milford, MA, USA). Polymeric film samples, encapsulated in an aluminum sample holder, were used. The monitoring of the measurement was performed under a nitrogen flow of 50 mL/min at a heating rate of 20 °C/min. All samples underwent a double sweep, the first one being from the selected starting temperature (50 °C) to a high temperature (375 °C) to eliminate traces of solvent and absorbed water. Subsequently, a second sweep was performed by heating the sample at a rate of 20 °C/min up to a final temperature (375 °C).

Wide-angle X-ray scattering (WAXS) was recorded at room temperature using a Bruker D8 discover A25 advanced diffractometer equipped with a Goebel mirror and using Cu Kα (*λ* = 1.542 Å) as the radiation source (Bruker, Billerica, MA, USA). The system was operated with a LynxEye detector using a step-scanning mode ranging from 5° to 70° (speed of 0.5 s and a 2θ step of 0.020°). The preferential intersegmental distance (*d*) in the chain packing of the amorphous polymers was determined using Bragg’s Law according to Equation (3):

(3) n λ = d sen θ

where *θ* is the scattering angle.

Scanning Electron Microscopy (SEM) micrographs were taken on the cross-section of the samples, which were previously fractured cryogenically and Au-metallized, using a QUANTA 200 FEG ESEM (Thermo Fisher Scientific, Waltham, MA, USA) in order to examine the interaction between the continuous and dispersed phases. The SEM was operated at an acceleration voltage of 1.5 kV under high vacuum using the detection of secondary electrons method.

The mechanical properties of the membrane films (neat membrane, MMMs and TR-MMMs) were measured in an MTS Synergie-200 testing machine (MTS Systems, Eden Prairie, MA, USA) equipped with a 10 N load cell, at room temperature, to determine their applicability. Hence, rectangular pieces (5 mm width × 30 mm length) were cut from the membranes and subjected to a tensile load at 10 mm/min until fracture. The calculated fractional free volume, FFV, is:

(4) $$FFV=\;\frac{V^{i}\;-\;V_{0}^{i}}{V^{i}}\;i=\mathrm{HPA},\mathrm{PPN}$$

where $V^{i}$ is the total specific volume, while $V_{0}^{i}$ is the specific skeletal volume of the i-th phase (i= HPA, PPN). The skeletal volume for HPA can be estimated from its van der Waals volume as $V_{0}^{HPA}\;\approx \;1.3V_{w}^{HPA}\;\cdot \;V_{w}^{HPA}$, and, accordingly, $V_{0}^{HPA}$ can be evaluated by using molecular modeling according to the Materials Studio software (BioVia Dassault Systémes, San Diego, CA, USA). The HPA specific volume $V^{HPA}$ can be obtained from its density as $V^{HPA}\;=\;1/\rho^{HPA}$. Density was measured according to the Archimedes principle in a CP225 Analytical Balance from Sartorius (Sartorius, Göttingen, Germany) equipped with a density measurement kit. The samples were weighed in air and into high pure isooctane at room temperature. The average density from seven samples was obtained as:

(5) $$\rho^{HPA}=\rho_{C_{8}H_{18}}\frac{W_{air}}{W_{air}-W_{C_{8}H_{18}}}$$

where $\rho_{C_{8}H_{18}}$ corresponds to the isooctane’s density, $W_{air}$ corresponds to the sample weight and $W_{C_{8}H_{18}}$ stands for the weight of the sample when submerged in isooctane. Then, Equation (4) allows for the evaluation of the FFV for HPA.

The PPN specific volume can be evaluated as the sum of its skeletal specific volume $V_{0}^{PPN}$ plus the specific volume within the PPN pores $V_{p}^{PPN}$:

(6) $$V^{PPN}=V_{0}^{PPN}+V_{p}^{PPN}$$

$V_{0}^{PPN}$ was measured in an AccuPyc 1330 V2.04N (Micromeritics Instrument Corporation, Norcross, GA, USA). Thus, the skeletal volume of PPN is determined by gas displacement using the volume–pressure relationship of Boyle’s Law.

Helium is used as the displacement medium. The sample is placed in a sealed cup of a known volume (2.5 cm^3^). Gas is introduced to the sample chamber and then expanded into a second empty chamber with a known volume. The pressure observed after filling the sample cell and the pressure discharged into the expansion chamber are measured, and then the volume is calculated. Density was determined by dividing the sample weight by its volume.

Furthermore, $V_{p}^{PPN}$ is measured by CO_2_ adsorption–desorption at 0 °C (273.15 K) in the volumetric device Nova 4200 (Quantachrome, Boynton Beach, FL, USA). Samples were degassed at 125 °C for 18 h under vacuum before the CO_2_ adsorption measurements. By using again Equations (4) and (6), the value of the PPN FFV was attained.

Finally:

(7) $$FFV^{MMM}=\;\phi FFV^{PPN}\;+\;\left(1-\phi \right)\;FFV^{HPA}$$

This correlation between the fractions of free volume and the mass fraction of the filler (PPN), ϕ, assumes that there is no significant interaction between the filler and matrix.

Another procedure can be followed by assuming that the van der Waals volumes are additive:

(8) $$V_{W}^{MMM}=\;\phi_{V}\;V_{W}^{PPN}\;+\;\left(1-\phi_{V}\right)V_{W}^{HPA}$$

with $\phi_{V}$ being the volume fraction of the filler. Equation (8) allows for the evaluation of $V_{w}^{MMM}$ from $V_{w}^{HPA}$ and $V_{w}^{PPN}$, as evaluated by molecular modeling, and, correspondingly, $V_{0}^{MMM}$. Then, once $V_{0}^{HPA}$, $V_{0}^{PPN}$ and $V_{0}^{MMM}$ are known, we can obtain $V^{MMM}\;=\;1/\rho^{MMM}$ and $V^{HPA}\;=\;1/\rho^{HPA}$, and, finally, Equation (6) would allow for the determination of FFV. Note that this second method does not assume zero interaction by postulating additivity, as is the case in Equation (7), but only concerns the van der Waals volumes, as shown in Equation (8). Consequently, the first method would be referred to here as the non-interaction or ideal method, while the second one would be referred to here as interactive.

### 2.8. Gas Permeability Measurements

The pure gas permeabilities (H_2_, N_2_, O_2_, CH_4_ and CO_2_) of the membranes were measured at 35 °C and 3 bar of feed pressure using a constant-volume/variable-pressure device. The membranes were placed on a brass plate with a 0.5 cm^2^ feed side membrane area (8 mm diameter), which, in turn, was placed in a permeation cell. The membrane was initially maintained under a high vacuum overnight to remove any residual gas. Then, at time t = 0, one side of the membrane was exposed to 3 bar of pressure. The increase in pressure in the permeate side was recorded as a function of time, allowing for gas permeation until the steady state was reached. The pure gas permeability of the target compound i (Pi) was calculated using Equation 8, where P is typically expressed as “Barrer” (1 Barrer = 10^−10^ cm^3^ (STP) · cm/(cm^2^ · s ·cmHg) = 3.35·10^−16^ mol·m/(m^2^ · s · Pa)).

(9) $$P_{i}=\;\frac{V_{i}l}{p_{2}ART}\left[{\left(\frac{dp_{1}}{dt}\right)}_{ss}-\;{\left(\frac{dp_{1}}{dt}\right)}_{leak}\right]$$

Here, *V* is the downstream volume (cm^3^), *l* is the membrane thickness (cm), *p^2^* is the upstream pressure (bar), *A* is the membrane area (cm^2^), *R* represents the ideal gas constant, *T* is the absolute temperature (*K*), *(dp_1_/dt)_ss_* is the steady-state rate of the pressure increase in the downstream volume (mbar s^−1^) and *(dp_1_/dt)_leak_* corresponds to the rate of pressure increase in the downstream volume in the leak assay (mbar s^−1^).

Finally, the ideal selectivity (α) for a gas pair was evaluated according to Equation (9), where *P_A_* stands for the faster gas permeability and *P_B_* stands for the slower gas permeability.

(10) $$\alpha_{A/B}=\;\frac{P_{A}}{P_{B}}$$

Moreover, according to the solution diffusion mechanism customarily assumed for gas separation membranes [27], permeability is the product of diffusivity and solubility, and, thus, $\alpha_{A/B}=\left(D_{A}/D_{B}\right)\left(S_{A}/S_{B}\right)$.

## 3. Results and Discussions

### 3.1. Thermal Properties of the Membranes

An analysis of the thermal stability of the membranes allowed for the elucidation of the ideal conditions to which these copolyamides should be subjected in order to induce the thermal conversion and achieve the formation of polymeric structures containing a high percentage of benzoxazole units in the final TR material. On the basis of the results obtained by Soto et al. (2020) [16] on hydroxypolyamides, the thermal reorganization process involving two diamines of different natures (not TR-able or TR-able ones), which is our case, has been investigated. 

**Figure 4:**
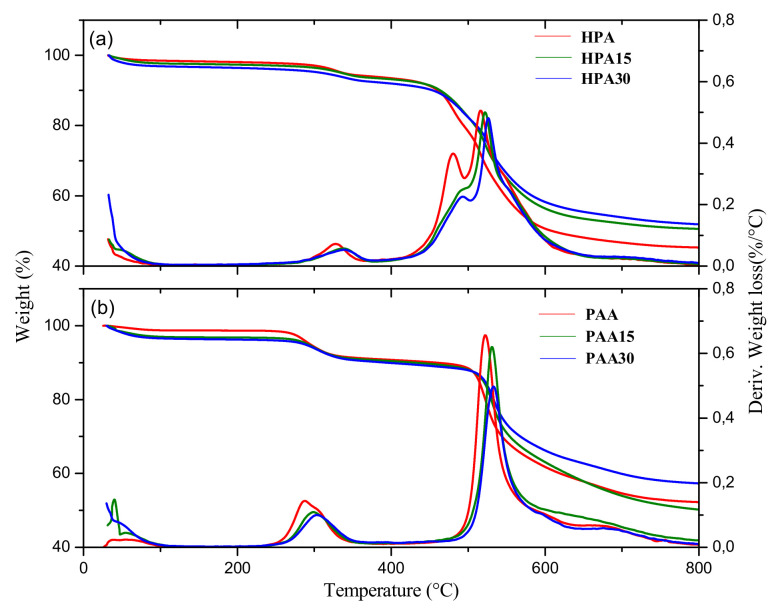
TGA curves of (**a**) HPA and (**b**) PAA and MMMs made from them, with PPN as the filler.

The TGA analyses of the two copolyamides and the MMMs showed two clearly differentiated stages (Figure 4). In the first step, in the range of 200 to 400 °C, the conversion of the precursor to TR-β-PBO occurred. This conversion was accompanied by a loss of the residual solvent that could not be removed during the previous heat treatment applied to the sample and of the water molecules per repeat unit (see Figure 3). The second step, above 450 °C, corresponded to the decomposition of the polybenzoxazole formed in the previous stage. At this point, it should be stressed that the kinetics of degradation of the TR-PBO derived from the copolyamide with 6FpDA were faster than those of the TR-PBO derived from the copolyamide with APA (Figure 4).

The conversion protocol of HPA and PAA to TR-PBOs was optimized by a detailed study using isothermal TGA analysis, following the procedure previously described elsewhere [16,28]. The weight loss of the HPA and PAA films (used as a conversion rate proxy) measured at the end of the first step (according to Equation (2)) was higher than the theoretical loss (i.e., 8.67% vs. 5.28% for HPA and 3.97% vs. 2.5% for PAA). This indicated that the solvent was not completely removed during the initial heat treatment for membrane drying, and a small amount of it remained trapped within the membrane. The highest sample conversion rate was achieved at 375 °C and 15 min (96% for PAA and 91% for HPA), which was determined via isothermal measurements at 375 °C for 120 min. In this context, Diez et al., (2018) [28] investigated the thermal conversion process for some polyamides similar to those used in the present work by evaluating the weight changes (via TGA), glass transition temperature (via DSC) and intensity of the band corresponding to PBOs (via ATR-FTIR) as a function of the temperature. These authors observed that the conversion of polyamides to PBO started at 250 °C, and the weight loss percentages were higher than the theoretical ones corresponding to the TR process (with the presence of the solvent). These results suggested that the thermal reorganization process started before the solvent removal, which depends on the polymer structure, and was likely higher for APA derivatives. Comparative TGA-FTIR studies were performed for the same family of copolyamides used in the present work. These studies concluded that the only difference in the conversion process to benzoxazole in the co-polymer HPA was the slightly lower temperatures compared to that of the co-polymer PAA. In this case, the quantitative formation of the TR-PBO derivative was reached at around 350 °C.

The thermal conversion of the TR-PBO structures in the MMMs was also investigated by isothermal TGA measurements. The same behavior observed for the neat membranes was recorded herein. Therefore, it was hypothesized that, even at the relatively high conversion temperatures for these materials, the solvent was still occluded. In the present work, the samples heated at 375 °C did not show any weight loss below 400 °C. The conversion process was confirmed by the presence of the benzoxazole bands at 1498 cm^−1^ in the FTIR spectra. Additionally, it was observed that the addition of PPN as a filler on the polymeric matrix enhanced the thermal stability on the MMMs, probably due to the high degradation temperature of the PPN (Figure 4).

The glass transition temperatures (Tg) of the neat membranes, MMMs and their corresponding PBO-TR were determined via DSC. Table 2 summarizes the changes in the Tg of the PAA- and HPA-based membranes. In our study, the Tg increased both with the addition of the filler to the polymeric matrix and after thermal rearrangement. Actually, there is a slight decrease in Tg for a high-enough (30% here) load. This initial increase in Tg is likely caused by the restriction of the molecular mobility of the polymer chains [29]. When the load of the filler increases ulteriorly, the filler could reduce rigidity and Tg by disturbing the chain packing of the matrix. This Tg versus filler content trend in composite materials has been consistently observed and reported in the literature [29,30]. Therefore, favorable polymer–filler interaction was hypothesized, as observed in the SEM images and in the resulting permeability (see Section 3.6). 

It is worth mentioning that Tg was quite similar for both HPA and PAA materials before thermal treatment. After thermal rearrangement, Tg was higher than it was before the thermal treatment, confirming a higher rigidity of the rearranged chains. Moreover, Tg was higher for PAA than it was for HPA after thermal rearrangement.

### 3.2. Chemical and Physical Properties of the Membranes

**Figure 5:**
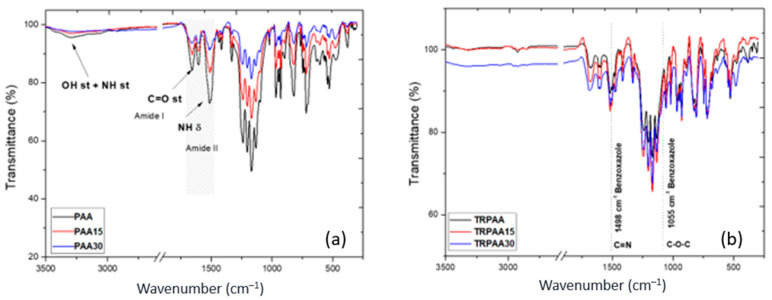
ATR-FTIR spectra (**a)** of neat membranes; MMM (15 and 30% of PPN) PAA and (**b**) HPA-based and their corresponding TR-PBO-MMMs.

The ATR-FTIR spectra of the copolyamides processed as polymer films were obtained to assign the characteristic bands of the functional groups in each structure and to confirm the presence of benzoxazole (PBO) units in the TR-MMMs. Neat membranes were used as a reference of the effect produced by the PPN loading. Figure 5a displays the infrared spectra of the PAA-based MMMs, and Figure 5b displays the spectra of the TR PAA-based MMMs.

The polyamide-amide-based membrane exhibited the principal absorbance bands of polyamides: the OH (st) + NH (st) stretching band (3300 cm^−1^), the symmetric and asymmetric stretching of C=O (1667 cm^−1^ and ~1598 cm^−1^) and the symmetric stretching band NH (δ) (~1516 cm^−1^) (Figure 5). Additionally, the N-H bending band at 1470 cm^−1^ from the PPN lactam rings was identified. The presence of the aliphatic band C-H st (2900 cm^−1^) was observed for both poly(o-hydroxyamide)s and TR-PBOs. The conversion from the *o*-hydroxyamide moiety to the benzoxazole one was confirmed by the decrease in the intensity of the amide carbonyl absorption band (1650 cm^−1^) in the normalized spectrum compared to the band at ~1200 cm^−1^, which remained unaffected during the conversion process. Moreover, the presence of the C=N st and C-O-O st at 1498 and 1055 cm^−1^, respectively, confirmed the conversion to TR-PBO. 

**Figure 6:**
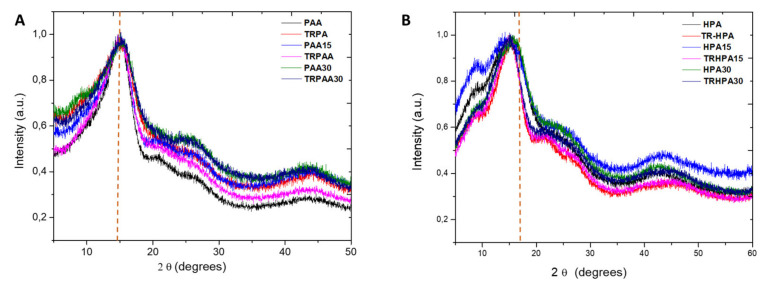
Diffractogram of MMMs and their corresponding TR from APAF-6FpDA-6FCl (**A**) and APAF-APA-6FCl (**B**).

The preferential intersegmental distances (referred to as d*_spacing_*) of membranes were obtained from WAXS spectra. Figure 6 compares the patterns of the copolyamides and TR copolyamide-based MMMs, including neat membranes (PAA, HPA and their β-TR-PBOs), as a function of 2θ. All samples exhibited the typical broad halo characteristic of their amorphous nature, with a maximum around 15°, which corresponds to a d*_spacing_* of ~0.59 nm. The most probable intersegmental distance, d*_spacing_*, along with the corresponding densities of neat membranes and MMMs, are shown in Table 3.

In principle, there could exist some correlation between d*_spacing_* and the packing density of the membrane. The HPA-based MMMs presented a peak at smaller angles (longer intersegmental distances) than those appearing for the PAA-based membranes. For example, the maximum d*_spacing_* was 0.52 nm (16.7°) for PAA30, and it was 0.57 nm (15.3°) for HPA30. Moreover, the same behavior was observed in the patterns of thermally treated membranes, with a larger difference in d*_spacing_* for TRPAA30 (16.1°, δ_max_ 0.54) compared with TRHPA30 (14.7°, δ_max_ 0.60 nm). These data demonstrated that the addition of PPN as a filler to polymer matrixes does not lead to any substantial change in the packing density of membranes. In all cases, thermal rearrangement gives slightly longer intersegmental distances. In accordance with the typical polymer behavior, an increase in the intersegmental distance should lead to an increase in the gas permeability [31,32]. It is also known that the presence of bulky pendant groups in the backbone chain of polymers typically increases d*_spacing_*, which supports an increase in gas permeability while maintaining selectivity if there is an increase in chain stiffness [32]. However, Shimazu et al. (2020) reported that d*_spacing_* does not always correspond to the intermolecular distance governing the diffusivity or permeability of the gas [33]. Actually, in the present work, the introduction of the bulky pendant groups increased d*_spacing_*, but the TR HPA-based MMMs with the largest intermolecular distance showed lower gas permeabilities (see Section 3.6 below) than the TR PAA-based MMMs with the smallest intermolecular distance.

### 3.3. Morphological Properties of the Membranes

The dispersion of the filler in the polymer matrix and the particle–polymer interface of the MMMs and their TR-PBO-MMMs were investigated by Scanning Electron Microscopy (SEM) from the cross-section of samples previously cryogenically fractured. This cryogenic fracture entails a vein-like morphology in the tested membranes induced by the influence of the filler on the conformation and packing of the polymer chains during the evaporation of the solvent [16].

**Figure 7:**
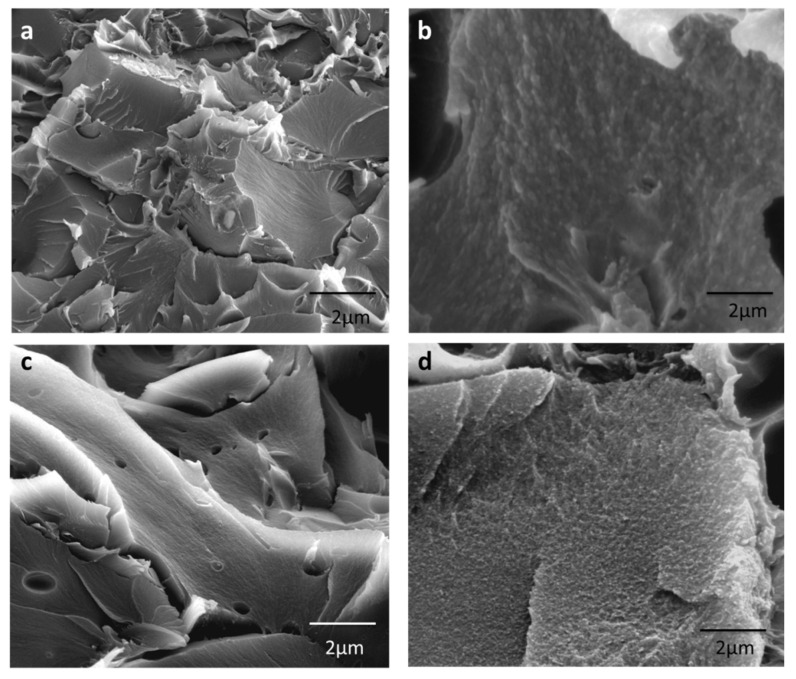
SEM micrographs of MMMs and their corresponding TR-PBO: (**a**) HPA30, (**b**) TRHPA30, (**c**) PAA30 and (**d**) TRPAA30. In any case, real pores cannot be confused with non-interconnecting grooves or cavities probably caused by solvent liberation.

SEM images of both MMMs and TR-PBOMMMs based on HPA and PAA as a polymeric matrix at 30% PPN loading are shown, as an example, in Figure 7. Both copolyamides exhibited a homogeneous dispersion with PPN particles, regardless of the filler load tested, which suggested a good compatibility between the matrix and PPN particles. No defects were observed on the interface, even for the 30 wt.% filler content (Figure 7a,c). It was hypothesized that the homogeneous dispersion and good adhesion of the target filler and the polymer matrix prevented agglomeration and the subsequent formation of non-selective channels and pore blockage defects (normally appearing during or after membrane manufacture). In addition, the observed increase in gas permeabilities at increasing filler loads supports the above-mentioned hypothesis (see Section 3.6). Gas permeability tends to decrease when the pores of the filler are partially blocked, while selectivity varies depending on the porous filler load [34,35]. On the other hand, the sieve-in-a-cage morphology, described by Moore and Koros (2005) [34], can mediate an increase in gas permeability without a significant decrease in the ideal selectivity, which could ultimately result in a favorable trade-off. In this context, stresses arising at the filler–matrix interface have been attributed to solvent evaporation.

On the other hand, slight changes in the morphology of the TR-PBO-based membranes were observed. Indeed, the cross-sections of these thermally treated membranes exhibited a smoother surface without deformation after thermal rearrangement for the tested copolyamides (Figure 7b,d). In addition, non-selective interfacial voids were recorded between the filler and the polymer matrix. It is worth pointing out that if these groves were intercommunicating both faces of the membrane, the selectivity would disappear completely. Finally, a similar increase in permeability without a significant impact on selectivity was observed in TR membranes (see Section 3.6 below).

### 3.4. Mechanical Properties of the Membranes

The data on the mechanical properties of the PAA- and HPA-based MMMs before and after thermal rearrangement are shown in Table 4.

It can be observed that the Young modulus, strain and maximum stress of the samples decreased after the addition of the PPN filler compared with the neat polymer. Moreover, the Young modulus decreases after thermal treatment. In general, thermal rearrangement increases both maximum stress and strain, although maximum stress is almost constant or decreases slightly for HPA after thermal rearrangement. In all cases, the mechanical properties are good enough, even after thermal treatment, although they are a little better for PAA before thermal treatment and for HPA after thermal rearrangement.

The mechanical properties of polymers depend on many parameters such as the casting procedure, temperature, molecular weight, etc. [31]. The results shown in Table 4 confirm the typical trade-off between mechanical properties and permeability (mechanical properties get worse with the increase in filler content in MMMs and after thermal rearrangement) and are comparable with those of some polymers reported in the literature [11,16,22,36,37,38,39]. In any case, the mechanical properties of this family of copolyamides (as a polymeric matrix) can certainly meet the requirements of gas separation processes.

### 3.5. Calculation of the Fractional Free Volume of the Membranes

**Figure 8:**
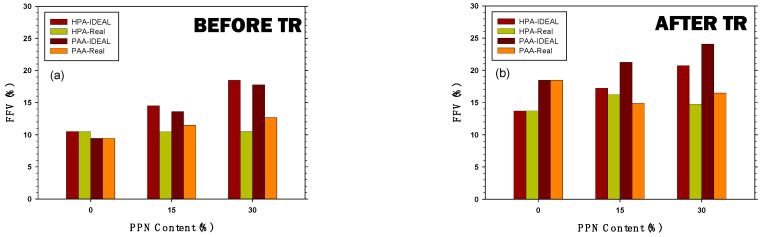
FFV as a function of the PPN content: (**a**) PAA and HPA before thermal rearrangement and (**b**) after thermal rearrangement. Ideal and interactive FFV calculations were explained in Equations (6) and (7).

The FFV corresponding to the MMMs studied, as obtained from both methods corresponding to Equations (7) and (8), are shown in Figure 8. It seems clear that FFV is higher after thermal rearrangement and increases with increasing PPN loads, in accordance with the permeability trends, as shown in Section 3.6. Moreover, the ideal FFV is lower for the PAA matrix MMMs before thermal rearrangement, and the tendency is inverted after thermal rearrangement. In turn, the interaction FFV is always quite similar for both polymeric matrices, although it also increases with PPN load and after thermal rearrangement. It thus seems clear that matrix–filler interactions, both before and after thermal rearrangement, decrease the FFV to be expected if this interaction was assumed as absent.

### 3.6. Gas Transport Properties of the Membranes

**Figure 9:**
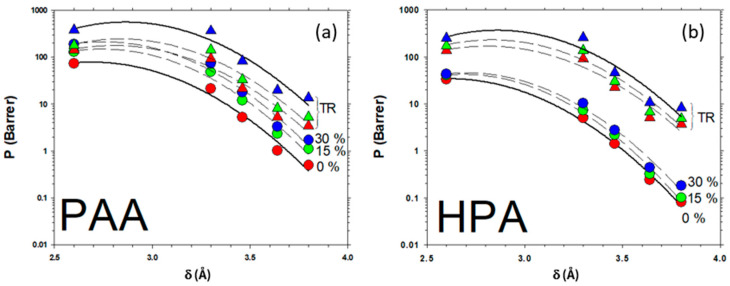
Permeability for the MMMs tested here as a function of the kinematic diameter of the permeated gas. For PAA (a) and (HPA (b). Note that 1 Barrer = 10^−10^ cm3 (STP) · cm/(cm^2^ · s · cmHg) = 3.35·10^−16^ mol · m/(m^2^ · s · Pa).

The gas transport properties of the neat membranes and HPA- and PAA-based MMMs were determined for He, O_2_, N_2_, CH_4_ and CO_2_ at 35 °C and 3 bar of feed pressure, before and after thermal rearrangement. The gas permeability of MMMs and the corresponding neat polymeric matrices of HPA and PAA are shown as a function of the kinetic diameter of the permeated gases, as given by Breck (1975) [40] for different filler contents, both before and after thermal rearrangement (represented in Figure 9). Recently, Soto et al. (2022) [25,41] proposed and validated a correlation between permeability and free volume, as:

(11) $$P=\;Ae^{B\;FFV}$$

With $B=\;a\;+\;b\delta \;+\;c\delta^{2}$; thus:

(12) $$\ln\;P=\;\left[\ln\;A+\;a\;FFV\right]+\;\left[b\;FFV\right]\delta \;+\;\left[c\;FFV\right]\delta^{2}$$

Here, *δ* is the kinetic diameter of the tested gas, and *A, a, b* and *c* are constants that, in principle, should be independent of the load.

In Figure 9, it can be seen that thermal rearrangement has a significant effect on the HPA copolymer, while, for PAA, the PPN load has similar effects as the thermal rearrangement. 

Equation (12) has been fitted in Figure 9, giving the parameters shown in Table 5.

From the values of b FFV and c FFV, we can obtain the FFV relative to the pure polymeric matrix both before and after thermal rearrangement that, assuming the interaction values shown in Figure 8 for the pure matrix membranes, gives the values of FFV shown in Table 6.

If we compare the values of FFV determined by permeability with those shown in Figure 8, it seems that gases permeate through the less compacted fraction of the membrane. 

In this context, gas permeability increased for all target gases with the addition of the PPN filler on the two copolyamides-based MMMs and TR-MMMs. The addition of PPN mediated a further increase in permeability for PAA-based MMMs higher than that for HPA-based MMMs. For instance, the addition of 15 wt.% PPN led to increases in membrane permeability for N_2_ and CO_2_ of 2.29-fold and 2.28-fold, respectively, whereas the Increase for HPA-based MMMs was only ~1.3-fold for N_2_ and ~1.4-fold for CO_2_, relative to the neat membrane PAA. Likewise, the PAA-based MMMs at 30 wt.% of the PPN load showed even greater permeability enhancements for N_2_ and CO_2_ (~2.3-fold for both gases) but a lower selectivity enhancement compared to HPA-based MMMs. Overall, despite gas permeabilities increasing with increasing PPN loadings, the selectivity was not significantly affected. Indeed, the selectivity CO_2_/CH_4_ and CO_2_/N_2_ in HPA15 only increased slightly compared to the pure membrane (70.6 and 22.5 versus 62.3 and 20.6, respectively).

The gas permeability of the copolyamide-based MMMs increased markedly after conversion to TR due to the loading of PPNs, which provided higher thermal stability. In this sense, PAA-based TR-MMMs again showed the largest increases in gas permeabilities. Thus, TR-PAA15 and TR-PAA30 presented larger increases in permeabilities for CO_2_ and N_2_ than TR-HPA15 and TR-HPA30. In addition, the pure copolyamides PAA and HPA showed a remarkable improvement in permeability after conversion to TR, presenting similar permeability values for all gases.

When comparing the permeability enhancements of the TR-MMMs with their corresponding enhancements of the pure TR membranes, the loading effect was larger for MMMs derived from HPA copolyamide, which could be caused by the low permeabilities of the polymeric matrix. For example, the CO_2_ permeability increased 28- and 52-fold for PPN loads of 15 and 30%, respectively, compared to pure TR membranes. Similarly, the CO_2_ permeability of PAA copolyimide-based MMMs was 7 and 17-fold higher when PPN was supplied at 15 and 30%, respectively, compared to the pure TR membrane. However, the effect of PPN loading on gas permeability was notably higher in PAA-based MMMs. Hence, CO_2_ permeability reached 363 Barrer for TR-PAA30 versus 259 Barrer for TR-HPA30. 

According to the membrane intersegmental spacing obtained by WAXS, the MMMs with bulky groups such as CF_3_ (HPA) were expected to support higher permeabilities for MMMs before thermal rearrangement, especially for CO_2_. In effect, bulky groups such as (C(CF_3_)_2_) could act as molecular spacers and chain stiffeners in the polymer, thus tending to increase the stiffness of the chains, leading to a reduction in intrasegmental mobility and limiting the degree of packing [5]. In addition, the CO_2_/CH_4_ selectivity was relatively higher for this family of membranes before and after thermal rearrangement, which suggests a positive effect of the filler on the polymer matrix. In this context, several criteria have been proposed in the literature to tune the transport characteristics of materials to form high performance MMMs for gas separation such as material selection. Thus, the polymeric matrix determines the minimum membrane performance, and the filler improves the ideal selectivity [34,35,42,43]. In the present work, it was hypothesized that the manufacture of MMMs from polymers capable of producing benzoaxazoles and with relatively low permeabilities might induce high gas permeabilities after the conversion to TR, since the dispersed phase would not compete with the continuous phase or vice versa. Therefore, it can be ruled out that the low permeabilities recorded were due to a poor compatibility between the particles and the polymer, and it can be assumed that this effect was purely due to the polymer backbone. Overall, the selectivities for all gas pairs were not significantly affected by the increase in permeabilities, which has been previously reported in the literature for MMMs [35].

**Figure 10:**
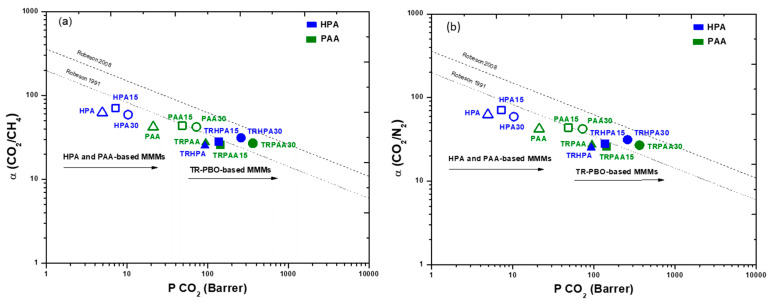
Upper bound limit plot for (**a**) CO_2_/CH_4_ and (**b**) CO_2_/N_2_ separation by MMMs based on PAA (solid symbols, green) and HPA (solid symbols, blue). The filler contents were 0 weight % (triangle), 15 weight % (square) and 30 weight % (circle). Lines correspond to the 1991 and 2008 Robeson upper bounds [44,45]. (a) corresponds to the CO_2_/CH_4_ gas pair and (b) corresponds to the CO_2_/N_2_ pair. Note that 1 Barrer = 10^−10^ cm^3^ (STP) · cm)/(cm^2^ · s · cmHg) = 3.35·10^−16^ mol·m/(m^2^ · s · Pa). Measurements were performed at 35 °C and 3·10^5^ Pa.

The results presented above were assessed via Robeson plots in Figure 10. It is worth mentioning that the permeability increased roughly in the same proportion as the diffusivity for the CO_2_/CH_4_ and CO_2_/N_2_ gas pairs when PPN was supplied at 15 and 30 wt.% in PAA-based MMMs. Finally, the upper bound 1991 for the CO_2_/CH_4_ gas pair was exceeded at a PPN of 30% in TR-PAA30 and TR-HPA30.

## 4. Conclusions

With a view to manufacture high-performance MMMs, two copolyamides, to be used as polymer matrixes have been synthetized. They consist of ortho-hydroxypolyamide (HPA) or ortho-hydroxypolyamide-amide (PAA). These polymeric matrices were filled with different loads (15 and 30 wt.%) of a microporous network (PPN).

The SEM images of the manufactured MMMs seem to assess the existence of the good compatibility between the two phases by promoting the homogeneous dispersion of the filler, even at a relatively high load without the detectable agglomeration of the filler.

For all the membranes studied, the mechanical properties were good enough, even after thermal treatment, although they were a little better for PAAs before thermal treatment and for the HPA after thermal rearrangement. The thermal stability was good, while Tg was higher for the PAA membranes than it was for the HPA ones. The rigidity of the chain, according to Tg, seemed to increase after thermal rearrangement and with increasing PPN loads.

The WAX results show that the addition of the PPN filler up to 30% does not substantially change the intersegmental distance. For all cases, the thermal rearrangement of the MMMs gave slightly longer intersegmental distances. It was observed that HPA-based MMMs exhibited slightly longer average intersegmental distances.

It appears that, for all the membranes studied, the free volume that determines gas transport is at the high end of the range evaluated, assuming either filler–matrix interaction or the absence of any interaction. This would mean that gas flux occurs mostly in the voids formed in the interface between the polymeric matrix and the filler. The FFV turned out to be quite similar for both HPA and PAA membranes, with some advantage for PAA after thermal treatment and for HPA before it. In all cases, FFV mostly increases by PPN addition and by thermal rearranging. Longer intersegmental distances seem to be more relevant for transport when the chain is somehow rigidified, as it happens after thermal rearrangement, giving higher permeabilities that are quite similar for both polybenzoxazole and poly(benzoxazole-amide) membranes. Before thermal rearrangement, PAA membranes give higher permeabilities despite having shorter intersegmental distances.

In general, both copoly(*o*-hydroxyamide)- and copoly(*o*-hydroxyamide-amide)s-based MMMs exhibited a notable improvement in gas permeability due to the presence of PPN for all gases tested, particularly for CO_2_. Moreover, after the addition of the filler, the selectivity was maintained for all loads, which could confirm the existence of a good compatibility of the copolyimide phase and the filler. 

In summary, although the thermal stability of the PAA seemed to be partly limited by the stability of the non-ortho-hydroxy polyamide chain, their mechanical properties were better. In addition, the permeability was higher for the PAA membranes before its thermal rearrangement and increased after the addition of moderate amounts of PPN. Finally, the high PPN-loaded MMMs surpassed the 1991 Robeson limit for CO_2_/CH_4_. The results obtained suggest that the incorporation of this filler on ortho-hydroxypolyamides offers a combination of good mechanical properties, a high thermal stability and an enhancement of gas transport properties.

## Figures and Tables

**Table 1:** Nomenclature of the studied membranes.

Continuous Phase	Neat Membranes	TR-PBO Membranes
Copoly-*o*-hydroxyamide-amide (APAF-6FpDA-6FCl)	PAA	TR-PAA
	PAA15	TR-PAA15
	PAA30	TR-PAA30
Copoly-*o*-hydroxyamide (APAF-APA-6FCl)	HPA	TR-HPA
	HPA15	TR-HPA15
	HPA30	TR-HPA30

15 and 30 represent the load percent of PPN in the membrane; HPA and PAA indicate that the MMMs are manufactured from o-hydroxy-polyamide or poly-o-hydroxyamide-amide, respectively; TR denotes thermally rearranged membranes.

**Table 2:** Tg of the PAA and HPA membranes before and after thermal treatment as a function of PPN content.

Tg (°C)		Tg (°C)	
PAA	283	TRPAA	304
PAA15	287	TRPAA15	306
PAA30	292	TRPAA30	304
HPA	279	TRHPA	296
HPA15	290	TRHPA15	298
HPA30	288	TRHPA30	293

**Table 3:** Comparison of density and d*_spacing_* for polyamides.

Polymer	Density (g/cm^3^)	d*_spacing_* (nm)	Polymer	Density (g/cm^3^)	d*_spacing_* (nm)
PAA	1.49	0.53	HPA	1.42	0.58
PAA15	1.41	0.53	HPA15	1.39	0.57
PAA30	1.35	0.52	HPA30	1.36	0.57
TR-PAA	1.44	0.55	TR-HPA	1.42	0.59
TR-PAA15	1.44	0.54	TR-HPA15	1.33	0.59
TR-PAA30	1.35	0.55	TR-HPA30	1.32	0.60

**Table 4:** Mechanical properties of the pristine and MMM studied.

(APAF-APA-6FCl) + PPN	Maximum Stress (MPa)	Strain (%)	Young Modulus (GPa)
HPA	84 ± 7	4.8 ± 0.4	2.3 ± 0.1
HPA15	51 ± 15	3.7 ± 1.1	1.8 ± 0.1
HPA30	55 ± 15	3.3 ± 0.7	2.0 ± 0.2
TRHPA	68 ± 12	6.7 ± 2.1	1.4 ± 0.2
TRHPA15	52 ± 17	4.4 ± 1.6	1.5 ± 0.1
TRHPA30	22 ± 18	2.3 ± 1.2	1.2 ± 0.3
**(APAF-6FpDA-6FCl) + PPN**	**Maximum Stress (MPa)**	**Strain (%)**	**Young Modulus (GPa)**
PAA	71.4 ± 3.6	4.7 ± 0.7	2.0 ± 0.2
PAA15	60.6 ± 9.9	4.6 ± 0.7	1.7 ± 0.1
PAA30	37.6 ± 7.7	3.2 ±0.7	1.4 ± 0.1
TRPAA	88.6 ± 7.9	10.1 ± 1.6	1.4 ± 0.1
TRPAA15	62.5 ± 12	5.7 ± 1.5	1.5 ± 0.1
TRPAA30	49.3 ± 5.6	5.4 ± 0.9	1.2 ± 0.1

**Table 5:** Constants in Equation (12) fitted to Figure 9.

		*ln*A + *a* FFV	*b* FFV	*c* FFV
Before TR	PAA	−11.65 ± 1.5	10.1 ± 1.5	−1.9 ± 0.3
	PAA15	−12.95 ± 2.9	11.0 ± 1.7	−2.0 ± 0.3
	PAA30	−12.74 ± 2.8	11.0 ± 2.8	−2.0 ± 0.4
After TR	TRPAA	−12.39 ± 4.1	10.5 ± 4.1	−1.9 ± 0.4
	TRPAA15	−13.37 ± 4.8	11.1 ± 1.8	−2.0 ± 0.5
	TRPAA30	−13.82 ± 5.0	11.6 ± 1.8	−2.0 ± 0.5
Before TR	HPA	−11.61 ± 2.7	10.1 ± 1.8	−1.9 ± 0.5
	HPA15	−14.01 ± 3.4	11.7 ± 2.0	−2.1 ± 0.4
	HPA30	−13.40 ± 2.9	11.2 ± 1.7	−2.1 ± 0.4
After TR	TRHPA	−12.28 ± 3.5	10.4 ± 1.7	−1.8 ± 0.4
	TRHPA15	−13.39 ± 4.0	11.1 ± 1.7	−2.0 ± 0.5
	TRHPA30	−13.82 ± 4.1	11.6 ± 1.8	−2.0 ± 0.3

**Table 6:** Free volume fractions according to permeability.

		*f* (%)
Before TR	PAA	9.44
	PAA15	17.44
	PAA30	17.44
After TR	TR-PAA	18.46
	TR-PAA15	23.96
	TR-PAA30	27.46
Before TR	HPA	10.50
	HPA15	25.00
	HPA30	20.00
After TR	TR-HPA	13.71
	TR-HPA15	21.21
	TR-HPA30	24.71

## Data Availability

The data presented in this study are available on request from the corresponding author.
